# Compliment to the Memory of Sir Charles Bell

**Published:** 1844-04

**Authors:** 


*Compliment to the Memory of Sir Charles Bell.*—Sir Robert Peel has addressed the following letter to Lady Bell:

"*Whitehall, September* 4.

"*Madam:* I have had great pleasure in recommending to Her Majesty, that in consideration of the high attainments of your lamented husband, and the services rendered by him to the cause of science, a pension of one hundred pounds per annum for your life, shall be granted to you, from that very limited fund which Parliament has placed at the disposal of the Crown for the reward and encouragement of scientific labors.

"This pension, small in amount as it necessarily is, will perhaps be acceptable to you, as a public acknowledgment on the part of the Crown, of the distinguished merit of Sir Charles Bell.

"I have the honor to be, Madam, your faithful and obedient servant,

ROBERT PEEL."

[*Am. Jour. Dental Sci.*

